# Voluntary Folic Acid Fortification Levels and Nutrient Composition of Food Products from the Spanish Market: A 2011–2015 Update

**DOI:** 10.3390/nu9030234

**Published:** 2017-03-05

**Authors:** María Lourdes Samaniego-Vaesken, Elena Alonso-Aperte, Gregorio Varela-Moreiras

**Affiliations:** Department of Pharmaceutical and Health Sciences, Faculty of Pharmacy, CEU San Pablo University, Madrid 28668, Spain; l.samaniego@ceu.es (M.L.S.-V.); eaperte@ceu.es (E.A.-A.)

**Keywords:** folic acid, voluntary fortification, food composition database

## Abstract

Introduction. Folic acid (FA) is a synthetic compound commonly added for voluntary fortification of food products in many European countries. In our country, food composition databases (FCDB) lack comprehensive data on FA fortification practices and this is considered a priority research need when undergoing nutritional assessment of the population. Methods. A product inventory was collected and updated by visiting retail stores in Madrid Region, conducting online supermarket searches, and by the provision of food label information by manufacturers. Euro-FIR FCDB guidelines for data compilation and harmonization were used. Results. The FCDB, compiled between 2011 and 2015, includes FA as well as macro and micronutrient data from 338 fortified foodstuffs. As compared to previous FCDB updates (May 2010), 37 products have ceased to declare added FA in their labels, mainly yogurt and fermented milk products. The main food subgroup is ‘breakfast cereals’ (*n* = 95, 34% of total). However, the highest average FA fortification levels per recommended serving were observed in the ‘milk, milk products, and milk substitutes’ group at ≥35% FA Nutrient Reference Values (NRV, 200 µg, EU Regulation 1169 of 2011) (60–76.3 µg FA per 200 mL). Average contribution to the FA NRV per food group and serving ranged between 16%–35%. Conclusion. Our data show a minor decrease in the number of FA fortified products, but vitamin levels added by manufacturers are stable in most food groups and subgroups. This representative product inventory comprises the main FA food source from voluntary fortification in our country. It is therefore a unique compilation tool with valuable data for the assessment of dietary intakes for the vitamin.

## 1. Introduction

Folic acid (FA) is the synthetic form of an essential water-soluble vitamin generically regarded as folates or B9. It is involved in one-carbon metabolism, and it has been linked to lowering Neural Tube Defect (NTD) risk when taken as a supplement around the time of conception [1]. Folates are also naturally present in foods such as green vegetables, fruits, liver, legumes, and nuts. Women of childbearing age are strongly recommended to maintain an adequate folate status through diet and supplementation, although the strategy has been proven to be somewhat ineffective in lowering the risk of NTD in Europe due to a high percentage of unplanned pregnancies and the relatively low compliance with FA pharmacological supplementation [2]. With an aim to increasing women’s FA intakes because of its public health relevance, fortification policies have been implemented worldwide. At present, only voluntary fortification of food products with FA takes place in Spain and the rest of Europe, whereas more than 60 countries add FA to wheat flour and other cereal products in a mandatory fortification scheme, according to data from the Food Fortification Initiative [3]. Voluntary fortification, also known as “discretionary fortification”, is the addition of vitamins or minerals to foods at the discretion of the manufacturer in order to restore micronutrients, ensure the nutritional equivalence of substitute foods, and/or to enhance the nutritive value of a product. In this regard, FA addition is endorsed by the European Regulation 1925/2006 of the European Parliament and of the Council of 20 December 2006, on the addition of vitamins and minerals and of certain other substances to food [4], and Regulation 1169/2011 of the European Parliament and of the Council of 25 October 2011, on the provision of food information to consumers [5]. This last regulation lays down the levels of “significant” vitamin addition, the requirements for nutritional and health claims, as well as the Nutrient Reference Values (NRV) for different vitamins, including folate.

In the last few years, an increasing number of researchers have questioned whether Europe should consider implementing mandatory fortification with FA, since current strategies, such as supplementation campaigns, have not been successful in reducing NTD prevalence [2,6]. On the other hand, concerns about the effects of extra FA intakes in children and the elderly are still a major issue that delays the implementation of this population-wide measure [7,8]. Although the Mediterranean diet is naturally a good folate source, data show that the Spanish population is folate deficient [9] and, most remarkably, the Mediterranean Diet is moving towards a less healthy pattern. Traditionally, the Mediterranean diet is characterized by a high consumption of vegetable foods (fresh fruit, vegetables, legumes, wheat bread, and olive oil) and fish, and a low intake of meats (mainly poultry). The latest national data indicate that fresh fruit, legume, and vegetable intakes are decreasing [9], which is in agreement with the data from the Spanish Food Consumption Survey Panel. Approximately only 50%–58% of the adult population reach current FA recommended intakes [10].

Food Composition Tables and Databases (FCDB) are key tools for nutritional assessment of the population’s diet. However, they are usually outdated in terms of inclusion of fortified products [11]. Important efforts have been made so far in developing specialized and standardized FCDB such as the one for the EPIC Project (European Prospective Investigation into Cancer and Nutrition) [12], and the Spanish FCDB BEDCA (Base Española de Datos de Composición de Alimentos) [13], both in line with the EuroFIR (European Food Information Resource Network) guidelines for harmonization and interchangeability [14]. However, the number of fortified food items included remains somewhat limited. It has been estimated that fortified foods provide only 5%–8% of the total energy intake of the European population [8], even though, market availability in the last 10 years has been consistently increasing [11]. Interestingly, data from surveys on total intakes of micronutrients (including fortified foods) in Europe and the US show that small proportions of the population, particularly children, may exceed the Upper Intake Levels (UL) for FA [15,16]. However, current fortification practices do not appear to contribute appreciably to the risk of adverse effects derived from nutrient intake [17]. Many researchers have outlined the importance of monitoring fortification practices and consumption of fortified foods in order to continuously assess the efficacy and safety of vitamin and mineral addition to foodstuffs [18,19,20,21,22]. At present, voluntary fortification practices are not being strictly monitored. An important number of brands commercialize fortified products in Spain [23], and continuous market evolution (new product launches and formulation changes) implies fortification level variations for specific vitamins. Food consumption surveys rarely assess fortified food products’ potential impact because of the absence of updated data on these products in most commonly used food composition tables and databases [11]. Nonetheless, important efforts have been made to include them, namely the Enkid study, which assessed consumption of fortified products such as breakfast cereals in children and adolescents [24].

For all the aforementioned, since December 2007 we have been actively working on the development of a comprehensive FCDB including all available FA fortified products from the Spanish market [23]. Enhanced reliability and comprehensiveness of food composition tables has been identified as a key research need worldwide in the context of a rapidly changing food supply [25]. Therefore, in this article we present the main findings and trends from the latest database update comprising FA fortified products commercialized in Spain.

## 2. Materials and Methods

Database design and structure has been previously described [23]. Briefly, Microsoft Office Access^®^ 2003 software (Microsoft Co., Redmond, Washington, DC, USA) was used for designing a tailor-made relational database. The LanguaL™ food description thesaurus and EuroFIR guidelines were adopted, including food group classification schemes [26]. The FCDB update was conducted through a market and online survey from January 2011 to June 2015 based in the Madrid Region. The consumption of fortified foodstuffs in the Madrid Region is higher as compared to the national average consumption [27], and the region includes the Capital City and a highly urbanized area (Metropolitan Madrid). Therefore, food availability in Madrid may be considered to include all fortified food products that would be available throughout different regions of the country. Retail centres such as hypermarkets, supermarkets, and convenience stores were visited over a four week period each year of the study and were selected in accordance with their sales data [28]. In addition, commercial online stores were accessed for product search, which includes food product availability nationwide. Foods that declared FA on the ingredient list by the following terms: B_9_, folate, folacin, or FA were identified and listed as potential FCDB inclusions. Compiled data included macro and micronutrients per 100 g or mL, recommended serving size, nutritional and health claims, and a photograph of the product package. Once completed, quality control checks were applied to the FCDB numerical data to assess potential errors and inconsistencies in recorded data. FA fortification levels were assessed as percentage of FA NRV (200 µg, [5]) provided per recommended serving for each food group as described in our previous studies [21]. All values are expressed per 100 g of edible portion on a fresh weight basis, unless otherwise stated. Food and Agriculture Organization (FAO) Guidelines for checking food composition prior to publication of a user database were followed when applicable [29].

Results of macro- and micronutrient contents are presented as median and interquartile range for skewed variables. Variables were tested for normality using a Kolmogorov-Smirnov test. All statistical analyses were performed using SPSS Software (SPSS 20.0, SPSS Inc., Chicago, IL, USA).

## 3. Results

**Figure 1:**
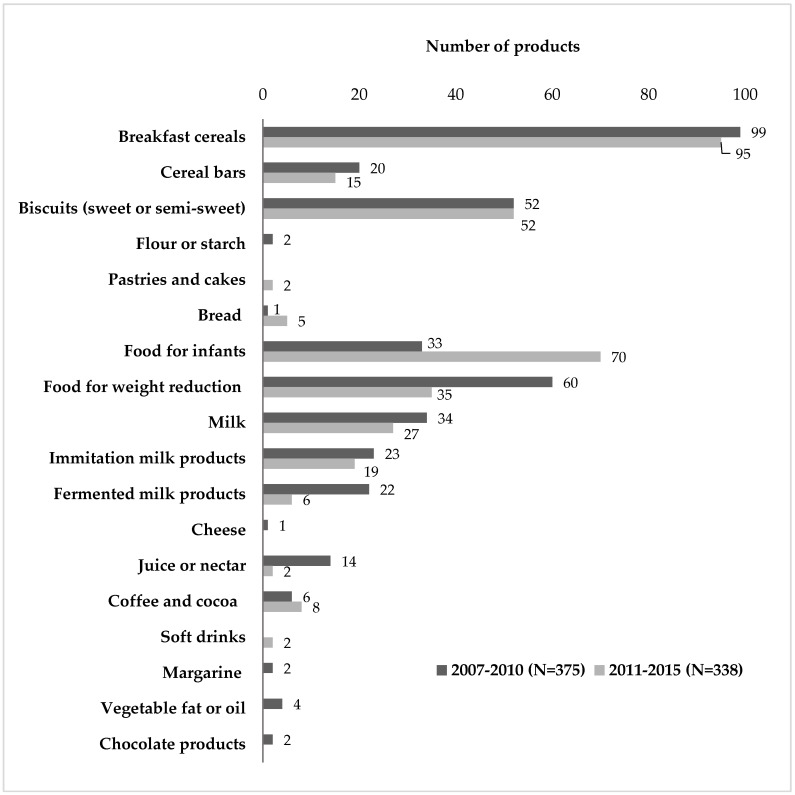
Food subgroup distribution in the Voluntary Folic Acid Fortification Food Composition Database: comparison between first (2007–2010) and second (2011–2015) compilation.

A total of 338 FA fortified products were compiled and assessed. 37 products were removed from the FCDB as they are no longer available for purchase or because FA is no longer included in their composition (Figure 1), mainly breakfast cereals, cereal bars, flours or starch, foods for weight reduction, milk, yogurts (fermented milks), cheese, juice or nectar, margarine and vegetable fats and oils, and chocolate products. A total of 25 products were new to the database, mainly foods for infants, coffee, tea, cocoa or infusions, soft drinks, pastries and cakes, and bread. Four food groups and 13 subgroups were included in the FCBD and their distribution is presented in Table 1. ‘Grain and grain products’ (50%, *n* = 169), and ‘products for special nutritional uses or dietary supplements’ (31%, *n* = 105) represented the highest proportion of available fortified products, while ‘milk, milk products, or milk substitutes’ (15%, *n* = 52) and ‘beverages (non-milk)’ (4%, *n* = 12) were minor. Total number of brands was 39, of which 31 were traditional manufacturer’s brands and 8 were from distribution (supermarket own brand). Ten products declared FA content on labels (ingredients list) but did not specify the quantity per 100 g or serving in the nutritional information label. Median declared FA contents ranged from 15 µg per 100 g (30 µg per 200 mL serving) in ‘nectar and juices’ and ‘soft drinks’ to 199 µg per 100 g (39.8 µg per 20 g serving) in ‘coffee, tea, cocoa, or infusions’ (Table 2). ‘Breakfast cereals’ is the subgroup that represented the highest proportion of FA fortified products (*n* = 95, 28% of total), with median declared FA levels of 170 µg per 100 g (51 µg per 30 g serving). Secondly, ‘foods for infants’ accounted for 20% (*n* = 70) of fortified products, with median declared FA levels of 65 µg per 100 g (16.25 µg per 25 g serving). Median energy content provided by each FA fortified food group ranged from 22.5 kcal per 100 mL in the case of ‘soft drinks’, to 406 kcal per 100 g in ‘cereal bars’. Carbohydrates were the main declared macronutrient in all groups, ranging from 5 to 80 g per 100 mL or g; of these, added sugars accounted for 5 to 72 g per 100 mL or g, and fibre content was 0.5 to 12 g per 100 g. Starch content, however, was not declared in most products. Total fat contents were 1.6 to 10 g per 100 g and the lipid profile (proportion of monounsaturated, polyunsaturated, and saturated fatty acids) was not declared in most food groups. Protein contents were 0.2 to 14 g per 100 mL or g.

A general outlook of the Access^®^ relational database, as well as an example of two database tables compiling fortified food data can be found at the Supplementary Material section. Most frequently added vitamins and minerals to FA fortified food formulations are shown in Table 2 and Table 3. All data are presented as medians and interquartile ranges. ‘Foods for infants’ and ‘foods for weight reduction’ were the groups that presented the highest proportion of simultaneous addition of vitamins other than FA, containing nearly all vitamins with the exception of vitamin K in ‘foods for weight reduction’. ‘Milk products’ declared to contain vitamins C, B1, B2, B3, B6, B12, pantothenic acid, and biotin; ‘imitation milk products’ contained vitamins A, D, E, and B6; ‘coffee, tea, cocoa or infusions’ declared vitamins A, E, D, C, B1, B2, B3, B6, B12, and pantothenic acid; and ‘breakfast cereals’ included mainly vitamins D, B1, B2, B3, B6, B12 and pantothenic acid, together with FA. In the case of added minerals, again ‘foods for infants’ and ‘foods for weight reduction’ were those that declared a higher number as compared to the other subgroups. All ‘grain products’ declared iron addition, and specifically ‘cereal bars’ and ‘bread products’ also declared the addition of calcium and sodium, respectively; some ‘milk products’ included calcium, phosphorus, and zinc addition. This shows that in most analysed products mineral addition was limited when compared to vitamins.

**Figure 2:**
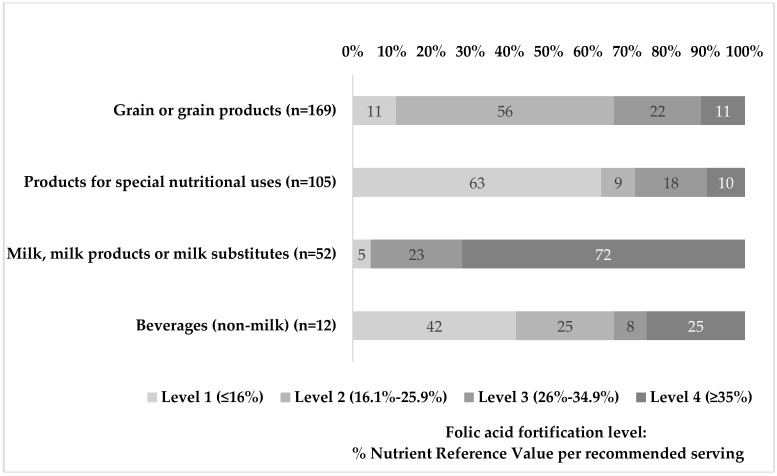
Folic acid fortification levels calculated per manufacturer’s recommended serving in compiled food groups from the Voluntary Folic Acid Fortification Food Composition Database.

FA fortification levels from compiled products were calculated from labelled FA values and recommended servings per each product, using the FA Nutrient Reference Value (200 µg/day, NRV) as guidance (Figure 2). In the major food group of the FCDB, ‘grain and grain products’, 56% of items presented fortification at level 2 which accounts for 16.1% to 25.9% of FA NRV per recommended serving. Fortification at level 1 (≤16% FA NRV) was observed in 63% of ‘products for special nutritional uses’ (‘foods for infant’ and ‘weight reduction’) and 72% of ‘milk, milk products or milk substitutes’ were fortified at the highest level available (level 4, ≥35% FA NRV). Finally, the ‘beverages’ group showed a higher proportion of level 1 fortification, as 42% of products provided less than 16% of FA NRV per recommended serving. These data should be interpreted with care in terms of FA contribution since NRV are set at 200 µg/day of FA according to EU Regulation, while in Spain, recommended FA intakes for women of childbearing age are 400 µg/day. Accordingly, the actual contribution of these products is well below half of this group’s needs.

## 4. Discussion

To our best knowledge, this is a unique updated food composition data compilation and assessment of FA fortified products available in the Spanish market. The Spanish fortified food supply is widespread through almost all available food groups. However, staple food products such as breads are only fortified to a limited extent when compared to other European countries such as Ireland and the UK, where breads, fat spreads, and fruit juices are commonly fortified [30]. In our country, fat spreads do not have added FA anymore, while only five years ago, up to six products containing FA were available [23]. Although it was not a main aim of our study, a limitation pertaining the use of the label-declared FA values is that we found fortification overages (higher than declared values added to products) in a number of products in previous studies [21]. The use of analytical FA contents from foodstuffs is always advisable, but the present work includes such a high number of products and types of food matrixes, that it would be a highly expensive and unaffordable task to undertake.

In order to assess the potential impact of FA-fortified products on a population’s nutritional status, it is also necessary to evaluate if product market availability is in line with the most-consumed products in our country. Although data on fortified food consumption in Spain are scarce, in 2011 a study by the Spanish Nutrition Foundation showed that fortified milks were the most consumed fortified product amongst the population (49.61 g/person/day) followed by fortified yogurt (14.22 g/person/day) [27] by using data of the Food Consumption Panel. Our data indicates that voluntary FA fortification strategies do not follow this trend as fortified ‘milk and milk products’ are decreasing over time, whilst ‘breakfast cereal’ availability remains constant.

The main challenges of voluntary food fortification are reaching target populations such as women of childbearing age, which should be the main objective, and avoiding extra FA intakes in non-target groups. In Spain, women of childbearing age are at risk of insufficient folate intakes since only 50% of recommended folate intake is ingested through diet at present [10]. The daily inclusion of fortified products could provide this population group with 20%–60% of FA recommended intake, when fortification level 4 products are consumed [31]. On the other hand, there is concern that population subgroups, including children and the elderly, may be at risk of consuming usual intakes above the UL. In a previous study we assessed the potential intake of the main FA fortified products by children aged 2–13 years and the ULs were exceeded in no case [32]. In addition, the composition of these foodstuffs has also been discussed previously, as a high percentage of them contain high levels of added simple sugars, salt, and fat [33]. According to our results, fortified groups contain between 2–24 g of sugar per 100 g in ‘bread and similar products’ and ‘breakfast cereals’. However, these quantities should be considered on the serving basis, since ‘breads’ may contain 1.2 g of sugar per 60 g serving and ‘breakfast cereals’, 7.2 g of sugar per 30 g serving (Table 1). Taking into consideration the World’s Health Organization (WHO) and the European Food Safety Authority (EFSA) recommendations to keep simple sugar intake below 10% of daily Total Energy Intake [34], especially amongst the child population, specific fortified food products could be included with moderation in the context of a varied diet. The nutritional benefits of increased vitamin intakes should not be outweighed, however, by the risks according to the present results. Whether voluntary fortification is beneficial depends on which foods manufacturers fortify, which nutrients are chosen as fortificants, how much of the fortificant is added, and what portion of the population consumes the fortified products.

For research purposes, databases must be constantly updated to reflect the rapidly evolving marketplace, so that the contribution of both added and intrinsic micronutrients may provide accurate estimates of population intakes. In 2007, Irish researchers examined the effect of voluntary food fortification on dietary intake and biomarker status of folate and other homocysteine-related B vitamins in a healthy adult population (aged 18–92 years) using an updated FCDB [22]. They found that red blood cell folate concentrations were 387 nmol/L higher and plasma total homocysteine concentrations were 2 µmol/L lower in the group with the highest fortified food intake (median FA intake: 208 µg/day), as compared to the non-consumers of fortified foods, showing a substantial increase in dietary intakes as well as biomarkers of folate status. Regardless the widespread availability of FA fortified products, this type of assessment has not been performed to date in a representative sample of the Spanish population. It would be advisable to have detailed food consumption data at a brand level from the Spanish population in order to evaluate the actual impact of the observed fortification practices with FA. Data is scarce and therefore quantification of the underestimation of FA intake due to the non-consideration of fortified products is still speculative. According to our previous studies, the non-consideration of FA fortification may underestimate as much as 40% of actual folate and folic acid intake in women [31] and children [32]. In the Irish study [22] the difference in total folate and FA intake between consumers and non-consumers of fortified products is above 50%. These observations should be taken into account if the availability of FA fortified food items prevails and may be consumed by a significant number of individuals.

## 5. Conclusions

This newly updated and representative database reflects the energy and nutrient composition data of FA voluntarily fortified foods from four main categories commercialized in Spain. There is an important number and variety of available products. Our data show a minor decrease in FA fortified products, but vitamin levels added by manufacturers are stable in most food groups and subgroups. It is therefore a unique compilation tool with valuable data for the monitoring and assessment of dietary intakes of this vitamin.

## Figures and Tables

**Table 1:** Macronutrient distribution, fibre and salt content in folic acid-fortified food products from the Spanish market.

Food Groups and Subgroups	*N*	Energy (kcal)	Declared Serving (g or mL)	Fats (Total) (g)	SFA (g)	MUFA (g)	PUFA (g)	Carbohydrates (g)	Sugars (g)	Starch (g)	Fibre (g)	Protein (g)	Salt (g)
**Grain or grain products**	169												
Breakfast cereals	95	386 (378–403)	30 (25–30)	3 (1–7)	1 (0–3)	ND	ND	76 (67–81)	24 (20–30)	ND	5 (3–8.25)	8 (6–8)	0 (0–1)
Biscuits, sweet and semi-sweet	52	444 (423.2–462.7)	25 (25–29.5)	15.5 (12–17.5)	2 (1–4)	ND	ND	66.5 (63–71.5)	21 (17.5–23)	ND	4 (2–7.75)	7 (6–8)	0.41 (0.23–0.83)
Cereal bars	15	406 (380–416.5)	25 (23–30)	10 (7–13)	4 (3–9)	ND	ND	67 (61–74)	30 (23–35)	0 (0–36.5)	4 (3–6)	6 (5–7)	ND
Bread and similar products	5	375 (368–389)	ND	4 (3–5.5)	0.6 (0.45–1.65)	1.25 (0.75–2.2)	2.5 (0.87–4.05)	63 (59–70)	2 (1–4)	ND	12 (6.5–14)	14 (12–15)	1 (0.5–1)
Pastries and cakes	2	383 (383–389)	40	13 (13–13.5)	ND	ND	ND	58	34 (34–36)	ND	8 (8–8.5)	4	0.51 (0.45–0.58)
**Products for special nutritional uses or dietary supplements**	105												
Foods for infants	70	387 (90–483.2)	25 (20–24.75)	3 (2–21.2)	1.3 (0.47–7.72)	7.9 (1.7–9.7)	3.2 (0.5–5.1)	60 (13–78)	25 (7.7–39.2)	ND	0.5 (0–4.25)	9 (2–10)	0.1 (0.025–0.3)
Foods for weight reduction	35	383 (368–462)	45 (20–63)	12 (10–17)	5 (3–11)	ND	ND	45 (37–55)	29 (14–34)	ND	5 (2–8)	24 (6–26)	ND
**Milk, milk products, or milk substitutes**	52												
Milk	27	46 (38–52)	250	1.6 (0.3–1.95)	1 (0.275–1.1)	0.1 (0–0.6)	0 (0–0.3)	4 (4–5)	4 (4–5)	ND	ND	3 (3–3)	0.13 (0.13–0.2)
Imitation milk products	19	52 (49–60)	250	1.9 (1.6–2.5)	0.56 (0.41–1.05)	1.2 (0.4–1.4)	0.2 (0.2–0.3)	5 (4–6)	5 (4–6)	ND	0 (0–0.225)	3 (2–3)	0.13 (0.1–0.18)
Fermented milk products	6	49 (45–77)	65 (65–100)	1.85 (0.32–2.2)	0.2 (0.17–0.37)	1.15 (0.17–1.4)	0.6	3 (3–12.7)	3 (3–12)	ND	1.1	2 (2–2.25)	0.1 (0.1–0.12)
**Beverages (non-milk)**	12												
Coffee, tea, cocoa, or infusion	8	386.5 (371–548.5)	20 (15.25–30)	3.5 (2.5–16)	1 (1–8)	ND	ND	80 (73.5–81)	72 (61.5–75)	ND	6.5 (4–8.5)	5.5 (4.25–35.25)	0 (0–2.25)
Juice or nectar	2	24	200	ND	ND	ND	ND	5.5	5.2	ND	0.4	0.2	0.025
Soft drinks	2	22.5 (22–22.5)	310	ND	ND	ND	ND	5	5	ND	ND	ND	ND
TOTAL	338												

*N* = number of products. Values are expressed as median and interquartile range per 100 g or mL. ND = not declared; SFA = saturated fatty acids; MUFA = monounsaturated fatty acids; PUFA = polyunsaturated fatty acids.

**Table 2:** Vitamin content distribution in folic acid-fortified food products from the Spanish market.

Food Groups and Subgroups	*N*	A (µg)	D (µg)	E (mg)	K (µg)	C (mg)	B1 (mg)	B2 (mg)	B3 (mg)	B6 (mg)	Folic Acid (µg)	B12 (µg)	Biotin (µg)	Pantothenic Acid (mg)
**Grain or grain products**	169													
Breakfast cereals	95	ND	0 (0–1)	0 (0–10)	ND	ND	0 (0–1)	1 (1–1)	13 (13–14)	1 (1–1)	170 (166–200)	2 (0–2)	ND	0 (0–5)
Biscuits, sweet and semi-sweet	52	ND	ND	ND	ND	ND	ND	ND	ND	ND	100 (71.5–100)	ND	ND	0 (0–1)
Cereal bars	15	ND	0 (0–2)	0 (0–4)	ND	ND	0 (0–1)	1 (1–1)	13 (11–16)	1 (1–1)	170 (140–200)	2 (0–2)	ND	2 (0–5)
Bread and similar products	5	ND	ND	ND	ND	ND	ND	ND	0 (0–4.5)	0 (0–0.5)	100 (0–100)	ND	ND	0 (0–1.5)
Pastries and cakes	2	ND	ND	ND	ND	ND	ND	ND	10	ND	126	1	ND	ND
**Products for special nutritional uses or dietary supplements**	105													
Foods for infants	70	375 (101–459)	7.5 (1.7–8.9)	4 (1.3–6.2)	5.8 (0–33)	25 (14–71.5)	0.4 (0.15–0.58)	0.6 (0.11–0.87)	4.5 (1.7–6.95)	0.34 (0.1–0.6)	65 (15–70)	0.5 (0.1–1.18)	12 (1.9–15)	2.8 (0.4–3)
Foods for weight reduction	35	375 (105–467)	2 (1–3)	5 (4–7)	ND	25 (0–36)	0.7 (0.44–0.97)	0.76 (0.37–1.1)	8.4 (5.5–12)	0.9 (0.7–1.2)	120 (76–150)	0.38 (0.7–1.2)	12 (8–33)	1.9 (0.9–3)
**Milk, milk products, or milk substitutes**	52													
Milk	27	120 (120–120)	0.75 (0.75–0.76)	1.8 (1.5–1.8)	ND	0 (0–4.5)	0 (0–0.085)	0 (0–0.105)	0 (0–0.8)	0 (0–0.105)	30 (30–30)	ND	ND	0 (0–0.45)
Imitation milk products	19	120 (120–120)	0.75 (0.75–0.8)	1.8 (1.5–1.8)	ND	0 (0–12)	0 (0–0.21)	0 (0–0.23)	0 (0–2.4)	0.24 (0.2–0.3)	30 (30–30)	0.38 (0–0.4)	0 (0–4.9)	0 (0–0.8)
Fermented milk products	6	ND	0 (0–0.75)	ND	ND	ND	ND	ND	ND	0.85 (0.2–0.9)	90 (30–145)	0 (0–0.38)	ND	ND
**Beverages (non-milk)**	12													
Coffee, tea, cocoa, or infusion	8	0 (0–716)	5 (5–7)	0 (0–13.75)	ND	48 (46–78)	1 (0–1)	0 (0–1)	18 (16.25–21)	1.5 (1–2)	199 (190–283)	0 (0–1.5)	ND	3 (1–5.5)
Juice or nectar	2	60 (0–60)	ND	ND	ND	6 (0–6)	ND	ND	1 (0–1)	ND	15 (0–15)	ND	3 (0–3)	ND
Soft drinks	2	ND	ND	ND	ND	ND	ND	ND	ND	ND	15 (0–15)	ND	ND	ND
TOTAL	338													

*N* = number of products. Values are expressed as median and interquartile range per 100 g or mL. ND = not declared.

**Table 3:** Mineral content distribution in folic acid-fortified food products from the Spanish market.

Food Groups and Subgroups	*N*	Sodium (mg)	Potassium (mg)	Calcium (mg)	Phosphorus (mg)	Magnesium (mg)	Iron (mg)	Zinc (mg)	Copper (mg)	Manganese (mg)	Selenium (µg)	Iodine (µg)
**Grain or grain products**	169											
Breakfast cereals	95	ND	ND	ND	ND	ND	7 (7–8)	ND	ND	ND	ND	ND
Biscuits, sweet and semi-sweet	52	ND	ND	ND	ND	ND	ND	ND	ND	ND	ND	ND
Cereal bars	15	ND	ND	0 (0–760)	ND	ND	7 (0–10)	ND	ND	ND	ND	ND
Bread and similar products	5	400 (0–480)	ND	ND	ND	ND	0 (0–3.5)	ND	ND	ND	ND	ND
Pastries and cakes	2	ND	ND	ND	ND	ND	6	ND	ND	ND	ND	ND
**Products for special nutritional uses or dietary supplements**	105											
Foods for infants	70	0 (0–139)	0 (0–507.7)	175 (121–372)	59 (0–228)	0 (0–38.2)	6 (2.1–7.5)	0.8 (0–3.8)	0 (0–0.3)	0 (0–0.525)	0 (0–8.95)	12 (0–74.7)
Foods for weight reduction	35	29 (0–91.5)	781 (267.5–1091)	328 (0–436)	367 (0–469)	88 (0–110)	8 (0–10)	5 (0–6)	0 (0–0.65)	0.47 (0–0.8)	28 (0–31.5)	61 (0–86)
**Milk, milk products, or milk substitutes**	52											
Milk	27	ND	ND	120 (110–160)	0 (0–120)	ND	ND	ND	ND	ND	ND	ND
Imitation milk products	19	ND	ND	120 (105–132)	0 (0–67)	ND	ND	0 (0–1)	ND	ND	ND	ND
Fermented milk products	6	ND	ND	ND	ND	ND	ND	ND	ND	ND	ND	ND
**Beverages (non-milk)**	12											
Coffee, tea, cocoa, or infusion	8	ND	0 (0–3132)	260 (145.2–1079)	301.5 (0–1091.5)	170 (0–236)	7.5 (0–24)	0 (0–11)	0 (0–0.75)	0 (0–0.75)	0 (0–45)	0 (0–139.5)
Juice or nectar	2	ND	ND	ND	ND	ND	ND	ND	ND	ND	ND	ND
Soft drinks	2	ND	ND	ND	ND	ND	ND	ND	ND	ND	ND	ND
TOTAL	338											

*N* = number of products. Values are expressed as median and interquartile range per 100 g or mL. ND = not declared.
