# Supplementary material for: Voluntary Folic Acid Fortification Levels and Nutrient Composition of Food Products from the Spanish Market: A 2011–2015 Update
Source: Nutrients. 2017 Mar 5;9(3):234. doi: 10.3390/nu9030234 (PMC5372897; doi:10.3390/nu9030234)
Supplement: Supplementary file 1 [file nutrients-09-00234-s001.docx]

**Supplementary material**

**Figure 1**. Access^®^ relational database outlook from the Voluntary Folic Acid Fortification food composition database.


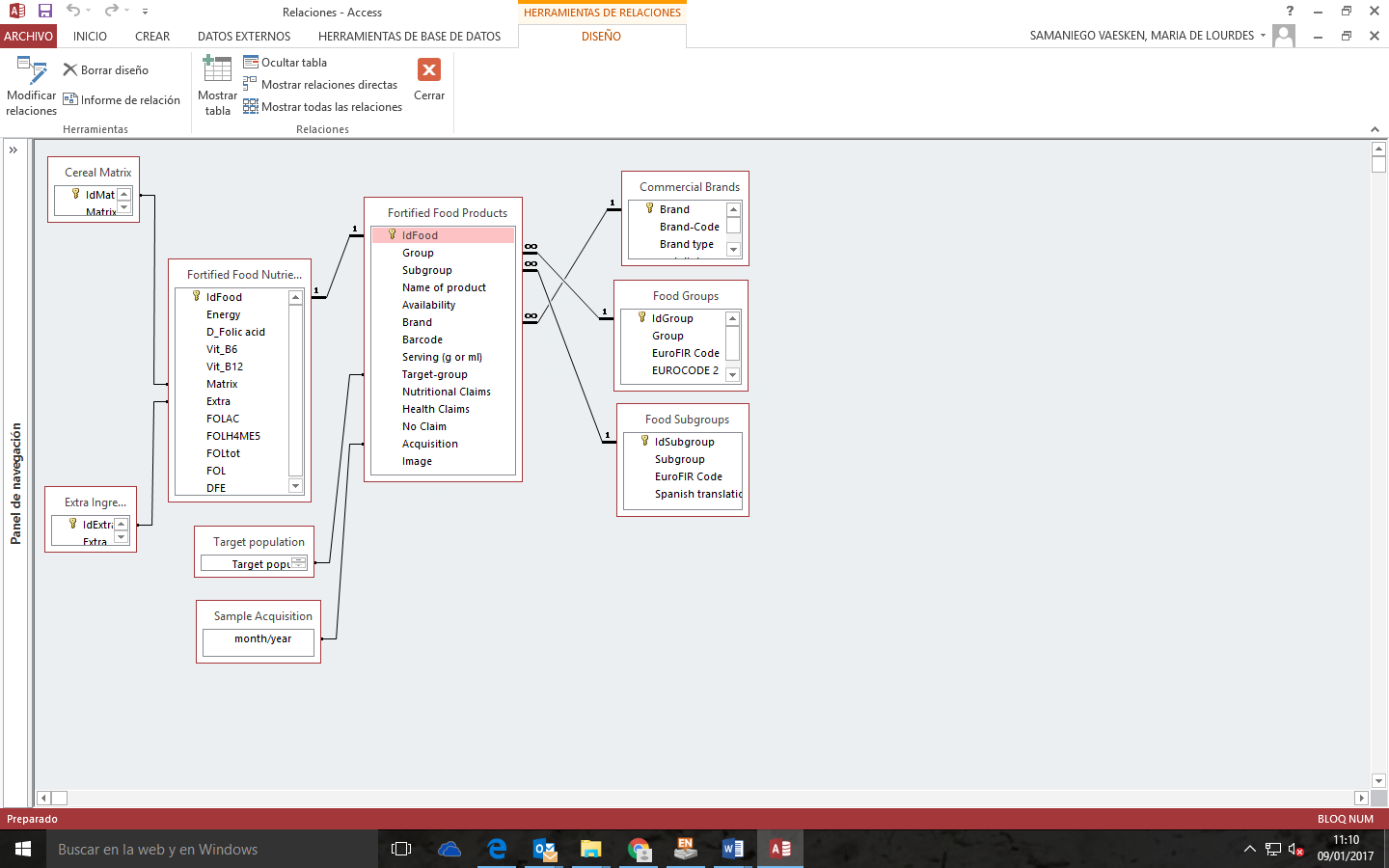


**Figure 2.** Screenshot taken from the Voluntary Folic Acid Fortification Food Composition Database: table compiling codification and general label information on food products.


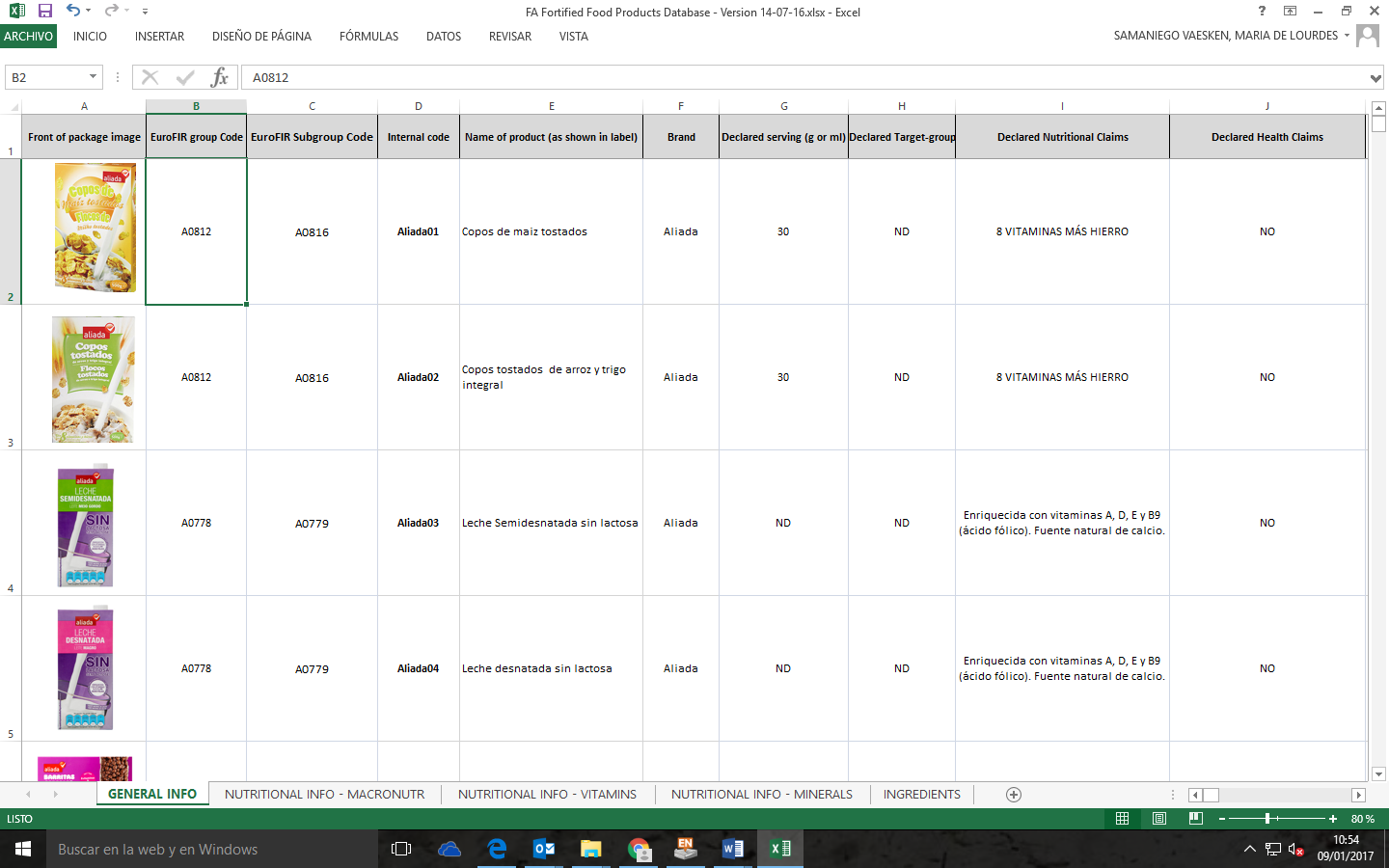


ND: not declared by manufacturer.

**Figure 3.** Screenshot taken from the Voluntary Folic Acid Fortification Food Composition Database: table compiling codification and nutrient label information on food products.


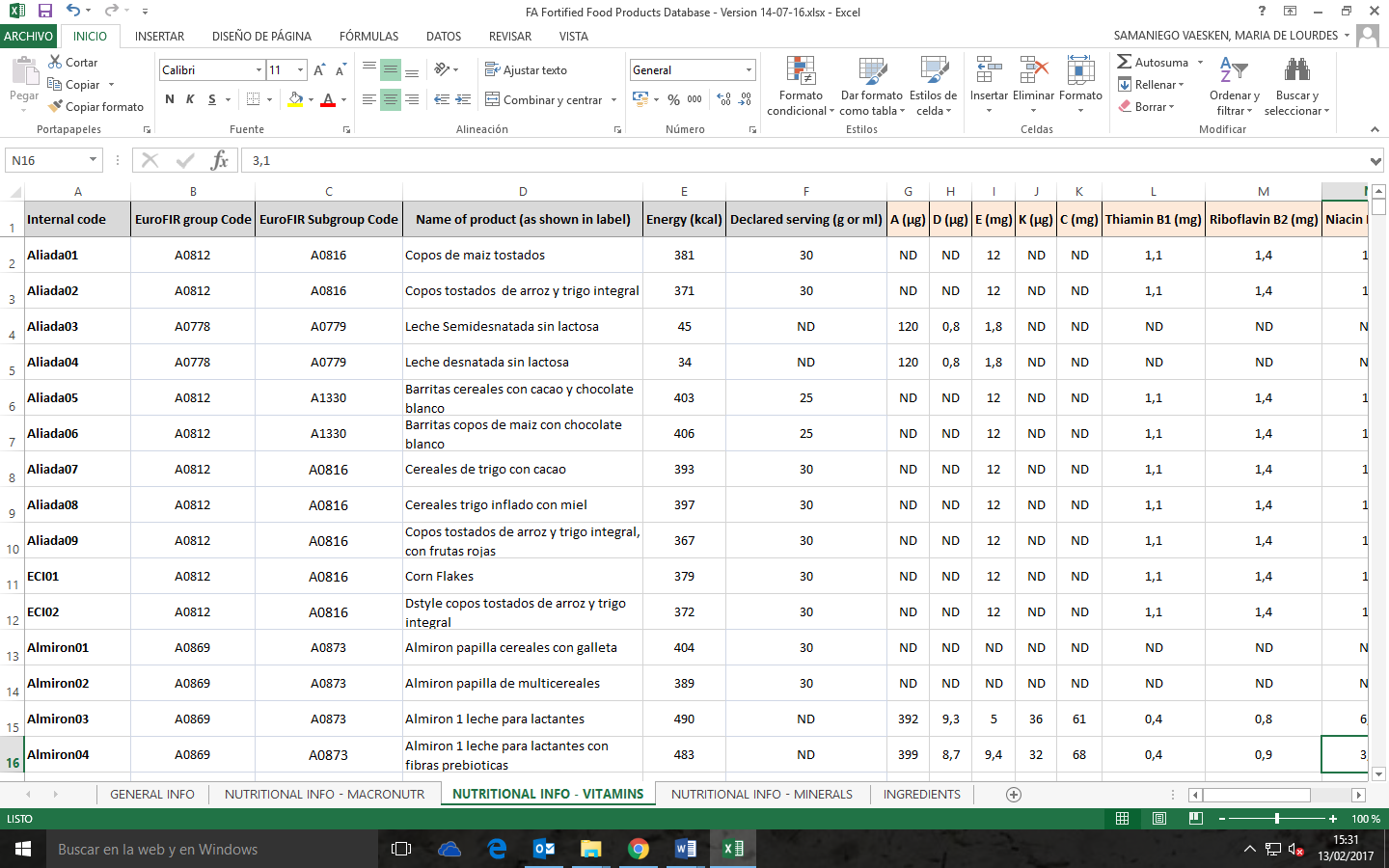


ND: not declared by manufacturer.
